# The anti-inflammatory and analgesic activities of 2Br-Crebanine and Stephanine from Stephania yunnanenses H. S.Lo

**DOI:** 10.3389/fphar.2022.1092583

**Published:** 2023-01-04

**Authors:** Lili Cui, Chaorui Peng, Jun Li, Xin Cheng, Xiao Fan, Jingyu Li, Zixian Yang, Yuancui Zhao, Yunshu Ma

**Affiliations:** ^1^ School of Pharmacy, Nanjing University of Chinese Medicine, Nanjing, China; ^2^ Yunnan Xinxing Occupations College, Kunming, China; ^3^ School of Chinese Material Medicine and Yunnan Key Laboratory of Dai and Yi Medicines, Yunnan University of Chinese Medicine, Kunming, China; ^4^ Key Laboratory of External Drug Delivery System and Preparation Technology in University of Yunnan Province, Kunming, China; ^5^ Yunnan Center for Disease Control and Prevention, Kunming, China; ^6^ Health Center of Majie Town of Yiliang, Kunming, China

**Keywords:** 10,11-dibrominecrebanine, stephanine, anti-inflammatory, analgesic, RAW264.7 macrophages

## Abstract

**Ethnopharmacological relevance:** Crebanine (Cre) and Stephanine (Step) are isoquinoline aporphine-type alkaloids that are extracted from *Stephania yunnanenses* H. S. Lo. Plants of the *Stephania* genus are often used for treatment of stomach pain, abdominal pain, and rheumatoid arthritis. Both Cre and Step exhibit strong activities but are also associated with a certain level of toxicity, 10,11-dibrominecrebanine (2Br-Cre) is a bromine-modified derivative of Cre that we prepared and tested in order to reduce toxicity and enhance efficacy.

**Aim of this study:** To investigate the anti-inflammatory and analgesic effects of 2Br-Cre and Step based on previous research findings and explore the specific biological mechanisms involved.

**Materials and methods:** The anti-inflammatory and analgesic effects of 2Br-Cre and Step were investigated using a range of experimental models, including xylene-induced ear edema, carrageenan-induced pleurisy, carrageenan-induced paw edema, the hot-plate test, the naloxone antagonism test and the acetic acid writhing test. A model of chronic constriction injury (CCI) of the sciatic nerve was also established to investigate therapeutic effects. A RAW264.7 cell model was established using lipopolysaccharide (LPS) to estimate the effects of these compounds on cytokines levels.

**Results:** 2Br-Cre and step significantly inhibited ear edema, paw edema and presented anti-inflammatory activity in the pleurisy model by inhibiting leukocyte migration and nitric oxide (NO) production, and by reducing the levels of PGE2. 2Br-Cre and Step significantly increased the pain threshold of mice subjected to heat stimulation; the effect was blocked by naloxone, thus suggesting that the analgesic effects of 2Br-Cre and Step were mediated by opioid receptors. 2Br-Cre and Step inhibited the frequency of writhing and prolonged the latency of writhing, and reduced the abnormal increase in the levels of BDNF in the serum and brain, thus alleviating the pain caused by CCI. In addition, 2Br-Cre and Step significantly inhibited the production of several inflammatory cytokines (IL-6, IL-1β and TNF-α) by LPS-induced RAW264.7 macrophages (*p* < .01).

**Conclusion:** 2Br-Cre and Step exerted remarkable anti-inflammatory and analgesic effects. As a structural modification of Cre, 2Br-Cre retains the anti-inflammatory and analgesic activity of Cre but with better efficacy. Consequently, 2Br-Cre should be investigated further as a lead compound for analgesia.

## 1 Introduction

The drugs used to treat inflammation fall into two broad categories: Steroidal anti-inflammatory drugs and non-steroidal anti-inflammatory drugs. Steroidal anti-inflammatory drugs are mainly steroid hormones and adrenocortical hormones, such as dexamethasone which can be used to treat pleuritis (Miyasaka and Mikami, 1982), cerebral edema (Rosa et al., 2016), otitis media (Dohar et al., 2006), and inhibit the levels of TNF-α, IL-1β and IL-6 (Zhou et al., 2022). However, These drugs can induce serious adverse reactions such as osteoporosis (Wu et al., 2022), immune deficiency (Dehghani et al., 2021) and infection (Kooistra et al., 2021). Non-steroidal anti-inflammatory drugs (NSAIDs) do not exhibit a steroidal structure and include aspirin and acetaminophen; these drugs exert anti-inflammatory anti-rheumatism effects (Koelz, 1994), pain relief (van Durme et al., 2021), antipyretic effects (Kazama and Senzaki, 2021) and anticoagulant effects (Villa Zapata et al., 2020), and are widely used for the treatment of osteoarthritis, rheumatoid arthritis and the relief of a variety of symptoms of fever and pain. NSAIDs predominantly exert anti-inflammatory effects by inhibiting the activity of cyclooxygenases (COXs), thus inhibiting the formation of prostacyclin (PGI_1_), prostaglandin (PGE_1_ and PGE_2_) and thromboxane A_2_ (TXA_2_) from arachidonic acid, thus inhibiting the aggregation of leukocytes, reducing the formation of bradykinin and inhibiting the agglutination of platelets. However, NSAIDs are also associated with serious adverse reactions, including gastrointestinal symptoms (Glowacka et al., 2022), renal toxicity (Moradi et al., 2021), hepatic toxicity (Hashizume et al., 2021) and anticoagulant effects (Dalgaard et al., 2020). Therefore, there is an urgent need to identify anti-inflammatory and analgesics drugs with new mechanisms of action and a reduced risk of side effects from natural products, such as traditional Chinese medicine.

As research on the anti-inflammatory effect of traditional Chinese medicine has advanced, it has become evident that some natural monomers of Chinese traditional herbs, single herbs and compound recipes of Chinese traditional herbs can exert good anti-inflammatory and analgesic effects by five main mechanisms. First, these herbs can regulate the hypothalamic-pituitary-adrenal (HPA) axis. The anti-inflammatory mechanism of Qiwei Tongbi (in an oral liquid form) was closely related to the pituitary-adrenal axis (Wang, 2010) while a combination of bovine keratin, baicalin, and baicalin showed therapeutic potential for atopic dermatitis by reducing the inflammatory response and attenuating activation of the HPA axis (Nguyen et al., 2020). Secondly, these herbs can affect the metabolism of arachidonic acid. Huanglian Jiedu Decoction was previously showed to relieve acute ulcerative colitis in mice by regulating the metabolism of arachidonic acid and glycerophospholipid (Yuan et al., 2020). Third, these herbs can influence the synthese of histamine, 5-HT and leukotriene B4. An extract prepared from the cortex of *Dichotoma chinensis* was previously showed to effectively inhibit the levels of histamine 5-hydroxytryptamine in ovalbumin induced acute inflammation in rats (Huang, 2008). Furthermore, the oral administration of taurocholic acidecerted obvious anti-inflammatory effects on adjuvant arthritis rats and significantly reduced the levels of LTB4 in the peripheral blood of rats (Li and He, 2008). Fourth, these herbs can influence immune regulation and the inhibition of leukocyte chemotaxis and activation. SI-NI-SAN was previouslyshowed to reduce the inflammatory cell infiltration of 2, 4-dinitrochlorobenzene (DNCB) induced atopic dermatitis auris, inhibit increased levels of cytokines in the serum, reduce the ratio of CD4+/CD8+T lymphocytes in the spleen, and alleviate skin dysfunction in mice by ecerting anti-inflammatory effects and regulating the immune system (Fan et al., 2018). Finally, these herbs can scavenging the free oxygen radicals that represent one of main pathological mechanisms in inflammatory reactions. When activated, Mcp triggers a burst of respiration that increases the levels of free oxygen radicals. Apigenin isolated from cockscomb was showed to scavenge free radicals and reversed the oxidative damage induced by a high-fat diet in rats (Dou et al., 2020).

Plants from *Stephania and Menispermaceae* are widely used in the area south of the Yangtze River in China. Most of these plants are able to dispel wind and relieve pain, clear heat and detoxify; they can also lead to sedation and induce diuresis to alleviate edema, these options are often used to treat stomach ache, abdominal pain, acute gastroenteritis, rheumatoid arthritis, malaria, carbuncle furuncle, swelling and eczema. For example, *Stephania tetrandra* S. Moore is a traditional Chinese herb that is mainly used to treat rheumatism and dysuria. *S. epigaea* H.S.Lo was referred to in the Materia Medica of Southern Yunnan written by Lanmao, a medical expert in the Ming Dynasty, and is capable of treating malaria, entorrhagia and carbuncle. Cepharanthine, extracted from *S. cepharantha* Hayata, is capable of increasing leukocyte levels (Suzuki et al., 1992) and exerts anti-tumor activity (Zhang et al., 2021) and can be used to treat the COVID-19 virus (Ohashi et al., 2021) and colitis (Wang et al., 2022a). These effects are mostly associated with the inhibition of NF-κB activation, lipid peroxidation, nitric oxide (NO) production, cytokine production and cyclooxygenase expression (Rogosnitzky et al., 2020). And are essential for viral replication and the inflammatory response. *Stephania yunnanenses* H. S. Lo has been used as an anti-inflammatory and analgesic medication (Lanmao, 1978) and to treat snake bites (Wang et al., 2022b).

Cre and Step are extracted from *Stephania yunnanenses* H. S. Lo, the tuberous root of which contains an abundance of isoquinoline alkaloids (Shen and Dong, 2016). Cre and Step exhibit a variety of structures and an extensive range of biological activities, including anti-inflammatory, analgesic, anti-arrhythmia, anti-tumor, antibacterial, anti-platelet aggregation, anti-hypertensive and immunomodulatory effects (chen, 2006; Wang et al., 2016; Zhao et al., 2021). Cre and Step have the same aporphine-type parent nuclear structure. Our previous study found that both Cre and Step could exert significant anti-arrhythmia and other biological activities (Wang et al., 2016) but also induced relatively high levels of toxicity in mice (Cre iv LD50 = 9.382 mg/kg; Step iv LD50 = 9.248 mg/kg) (Ma et al., 2005). It was previously reported that Cre could reverse the drug resistance of the leukemia drug-resistant cell line K562/HHT to enhance the anti-tumor effect of daunorubicin (Zhao and Shi, 2000), exerted obvious cytotoxicity to the human hepatocellular carcinoma cell SMMC-7721, and significantly inhibited the proliferation of hepatocellular carcinoma cells (Yang et al., 2010). Previous research has investigated the mechanisms responsible for the anti-inflammatory and anti-tumor activities of Cre. It is generally believed that the inhibition of NF-κB activity and its downstream gene expression profile reduces the TNF-α-induced proliferation, invasion and survival of cancer cells. Cre has also been shown to inhibit the production of pro-inflammatory cytokines (IL-6 and TNF-α) and the expression levels of iNOS and COX-2 in LPS-induced mouse macrophages (RAW264.7 cells), thereby inhibiting the production of NO and PGE2. This effect was closely associated with blockade of the NF-κB, Ap-1, Akt and mitogen-activated protein kinase (MAPKs) signaling pathways (Wang et al., 2022a). However, the pharmacokinetic data relating to the injection of Cre into rabbits showed that the half-life (t_1/2_) of Cre was short and was eliminated rapidly in the blood and tissues. The t_1/2_ during the distribution phase was 3.25 ± .22 min while that during the elimination phase was 36.67 ± 5.52 min (Ma et al., 2007). Moreover, both the therapeutic index and water solubility of Cre injection were relatively low. Collectively, these issues have serious effects on the medicinal properties of Cre. Therefore, our research group modified Cre at the structural level and obtained several derivatives, among them, 2Br-Cre present better anti-inflammatory activity and lower toxicity (Wang et al., 2016). From which 2Br-Cre was subsequently identified for further investigation. Due to the introduction of live bromine atoms, the toxicity of 2Br-Cre (mice LD50 = 59.62 mg/kg) was six-fold lower than that of Cre (Wang et al., 2016). Little is known about the precise pharmacological effects of Step and 2Br-Cre and their anti-inflammatory and analgesic pharmacological activities have yet to be reported.

In the present study, the anti-inflammatory and analgesic effects of 2Br-Cre (a structurally modified version of Cre) and Step (which has the similar parent nuclear structure as Cre) were investigated and compared using animal models and a RAW264.7 cell model.

## 2 Materials and methods

### 2.1 Reagents

Dexamethasone sodium phosphate injection (Dex) was purchased from Tianjin Suicheng Pharmaceutical Co., Ltd. (Tianjin, China). Zhengqingfengtongning Injection (Sin) was purchased from Zheng qing Pharmaceutical Co., Ltd. (Hunan, China). Naloxone (Nal) was obtained from Dalian Meilun Biotechnology Co., Ltd. (Dalian, China). Pethidine (PT) was obtained from Qinghai Pharmaceutical Factory Co., Ltd. (Qinghai, China). Rotundine sulfate injection (L-THP) was obtained from Guangdong Xinfeng Pharmaceutical Co., Ltd. (Guangdong, China). Carrageenan was obtained from Shanghai yuanye Bio-Technology Co., Ltd. (Shanghai, China). Cell Counting Kit-8 (CCK-8) was purchased from TransGen Biotech (Beijing, China). LPS was purchased from Sigma (St. Louis, MO, United States). ELISA kits for TNF-α, 1L-1β and 1L-6 were purchased from Proteintech Group Inc. (Chicago, America), FBS purchased from BI (Israel) while BDNF and 5-HTwere purchased from Sabbiotech (United States). RAW264.7 mouse macrophages were purchased from cell Resource Center, Huazhong University of Science and Technology (Guangzhou, China).

### 2.2 Herbal materials and the preparation of the 2Br-Cre and Step

**FIGURE 1:**
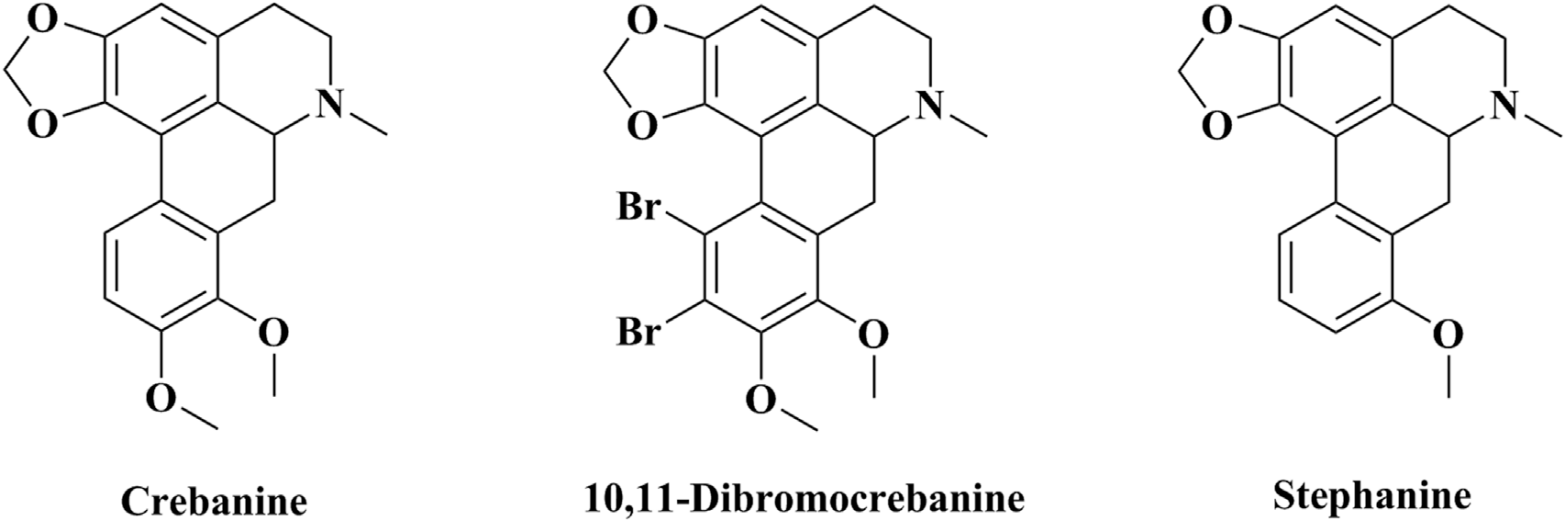
The chemical structure.

Plants (*Stephania yunnanensis*. H. S. Lo.) were collected from Yun County, Yunnan Province, China, and formally identified by Professor Yunshu Ma (College of Pharmaceutical Science, Yunnan University of Chinese Medicine). A specimen was deposited in the College of Pharmaceutical Sciences, Yunnan University of Chinese Medicine. Root tubers of *Stephania yunnanensis* H. S. Lo. Were used to isolated Cre and Step, as described previously (Chen et al., 1989; Zuo et al., 2013). Then, 2Br-Cre was obtained by modifying the structure of Cre, as described previously (Wang et al., 2016). 2Br-Cre was dissolved in 10% hydrochloric acid aqueous solution and the pH was adjusted to 6–7; this was then mixed with pure water. Step and Dex were also dissolved in pure water. Chemical structures are given in Figure 1.

### 2.3 Experimental animals

Kunming (KM) mice (20 ± 2 g) and Sprague-Dawley rats (180–220 g)were purchased from Hunan Skolek Jingda Experimental Animal Co., Ltd. [Reg. No. SCXK (Hunan) 2016–0002]. All animal protocols were approved by the Yunnan University TCM Committee on Animal Care and Use (Reference number: R-062016002) in the accordance with the National Institutes of Health guidelines for animal care. Animals were kept at 24°C ± 3°C with a 12 h dark/light cycle in a standard laboratory and fed a normal diet.

#### 2.3.1 Anti-inflammatory activities

##### 2.3.1.1 Xylene-induced ear edema

A model of xylene-induced ear edema was established in accordance with a previously published study (Wu et al., 2021). In total, 90 mice (20–22 g) were randomly divided into a Model group (NS, .2 ml), a Dex group (7 mg/kg), a Cre group (5 mg/kg), a 2Br-Cre group (with doses of 3, 6, and 12 mg/kg), and a Step group (with doses of .5, 1 and, 2 mg/kg), there were 10 mice in each group. Next, we applied xylene (20 μl) to the right ear of each rat with a pipette gun and applied an equal volume of saline to the left ear. Next, 20 min later, the drug treatment was administered *via* the tail vein. The mice were sacrificed 10 min later. Both ears were removed fromeach mouse and a punch biopsy (9 mm in diameter) were removed from each ears using a hole punch. The ear samples were then weighed accurately to determine degree of ear edema.

##### 2.3.1.2 Carrageenan-induced pleurisy

A model of carrageenan-induced pleurisy was established using previously described methodology (Kuraoka-Oliveira et al., 2020). In total, 72 SD rats (200–250 g) were randomly divided into a Model group (NS, 2 ml), a Dex group (4.83 mg/kg), a Cre group (3.45 mg/kg), a 2Br-Cre group (with doses of 2.07, 4.14, and 8.28 mg/kg) and a Step group (with doses of .34, .69, and 1.38 mg/kg); there were eight rats in each group. One hour after tail vein administration, we injected sterilized 1% carrageenan normal saline solution (.2 ml/100 g) between the sixth and seventh ribs of the right thoracic cavity of each rat. After 5 h, the animals were anesthetized and eutnanized. Next, we collected pleural exudate (20 μl) and diluted this to 600 μl with 1% acetic acid solution as a diluent to for leukocyte counting. In addition, the pleural cavity was washed with 1 ml of normal saline; then, we collected the exudate and rinse fluid. This fluid was centrifuged at 3000 rpm for 10 min and 100 μl of the supernatant was taken to determine the levels of PGE2 by isomerization. Then 100 μl of the supernatant was used to determine the levels of nitric oxide (NO) using a nitric oxide (NO) nitrate reductase kit in accordance with the manufacturer’s recommendations.

##### 2.3.1.3 Carrageenan-induced paw edema

A model of carrageenan-induced paw edema was established as described previously (Gou et al., 2017). In total, 90 mice (20–22 g) were randomly divided into a Model group (NS, .2 ml), a Dex group (7 mg/kg), a Cre group (5 mg/kg), a 2Br-Cre group (with doses of 3, 6, and 12 mg/kg) and a Step group (with doses of .5, 1 and 2 mg/kg). Next, 0.l ml 1% of carrageenan was subcutaneously injected into the right rear toe to cause inflammation. One hour after administration, the inflammatory swollen paw was cut off .5 cm above the ankle joint, weighed, and cut into small parts. The paw was soaked with 5 ml of normal saline for 1 h and then centrifuged at 3000 rpm. Then, we mixed .3 ml of supernatant and 2 ml of .5 mol/L KOH-methanol solution. After isomerization at 50°C for 20 min, the mixture was diluted to 4 ml with methanol and a UV-vis spectrophotometer (*λ* = 278 nm) was used to determine the levels of PGE2.

#### 2.3.2 Analgesic activity

##### 2.3.2.1 Hot plate test and naloxone antagonism experiment

The experiment was carried out by LS-6B intelligent hot-plate instrument, the temperature was 55°C ± .5°C, and the max exposure time was 60 s. Female mice (20 ± 2 g) were placed on a hot plate to measure the pain threshold. In total, 165 mice were randomly divided into 11 groups, including a normal saline group (NS, .2 ml), a PT group (25 mg/kg), a PT + Nal group (25 + 10 mg/kg), a 2Br-Cre group (12, 6, and 3 mg/kg), a Step group (2, 1, and .5 mg/kg), a 2Br-Cre + Nal group (12 + 10 mg/kg), a Step + Nal group (2 + 10 mg/kg), there were 15 mice in each group. Nal was injected intraperitoneally for 15 min and then administered via the tail vein. The basal pain threshold of each mouse was measured twice with an interval 5 min between each test. Then, the mean value was taken as the basic pain threshold. The pain threshold for each mouse was measured 15, 30, 60, 90, and 120 min after tail vein injection.

##### 2.3.2.2 Writhing test

In total, 80 mice were randomly divided into eight groups with 10 mice in each group (half male and half female). The groupings and administration doses were the same as that of mice in the hot plate test (Section 2.3.2.1). Twenty mins after tail vein administration, each animal was intraperitoneally injected with .6% glacial acetic acid (.1 ml/10 g weight). Then, we recorded the writhing times of each mouse over the next 20 min and writhing latency (the time of the first writhing reaction).

##### 2.3.2.3 Chronic constriction injury of the sciatic nerve

A model of chronic constriction injury (CCI) of the sciatic nerve was established as described previously (Bennett and Xie, 1988). In total, 110 rats were randomly divided into a Model Group (in which the sciatic nerve was isolated and ligated), a Sham Group (Only separation, no ligation), a positive group (L-THP, 13.8 mg/kg), a Cre group (3.5 mg/kg), a 2Br-Cre group (with doses of 2.1, 4.2, and 8.3 mg/kg) and a Step group (with doses of .45, .9, and 1.8 mg/kg). The Paw Withdraw Mechanical Threshold (PWMT) test was carried out on the rats in each group 1 day before modeling and 7 days after surgery to determine whether the modeling was successful. Starting on day 8, drugs were administered through the tail vein, Rats in the Model group and Sham group were injected with normal saline by the tail vein. The PWMT test was performed at 8, 10, 12, and 14 days after administration (for 30 min) and the PWMT was compared between each group and the Model group. Changes in body weight and behavior were observed before and after modeling. The levels of BDNF and 5-HT in the serum and BDNF in tthe brain were determined on day 14 after modeling.

### 2.4 Cell experiments

#### 2.4.1 Cell culture and cell viability

RAW264.7 cells (25×10^4^ cells/ml) were plated in 96-well plates (100 μl/well) and cultured at 37°C and 5% CO_2_ for 24 h. Then, the media was replaced and the cells in each well were treated with different concentrations of drug solutions dissolving with DMEM (2Br-Cre10, 20, 40, 80, and 160 μg/ml; Step .5, 1, 2.5, 5, and 10 μg/ml). After incubation for 24 h, 10 μl of CCK-8 solution was added to each well and the plate was further incubated at 37°C for 3 h. Finally, the absorbance (Abs) at 450 nm was determined.

#### 2.4.2 Nitrite assays and cytokine release assay

RAW264.7 cells were divided into several groups: a normal groups, a model groups (LPS, 1 μg/ml), a Dex group (50 μg/ml), a Sin groups (100 μg/ml), a 2Br-Cre group (80, 40, and 20 μg/ml) and a Step group (5, 2.5, and 1 μg/ml). RAW264.7 cells (25×10^4^ cells/ml) were plated in 96-well plates (100 μl/well) and cultured at 37°C and 5% CO_2_ for 24 h. The medium was replaced with msdia containing different concentrations of drugs for 2 h of pre-treatment, followed by the addition of LPS (1 μg/ml). After 9 h of incubation, the cell culture supernatant was extracted and stored at −80°C foranalysis. Then, the levels of TNF-α, IL- 1β and IL-6 were determined by using enzyme-linked immunosorbent assay (ELISA) kits in accordance with the manufacturer’s instructions.

### 2.5 Statistical analysis

All data were analyzed by SPSS version 22.0 and expressed as mean ± SD. significance was detemined by analysis of variance (One-Way ANOVA) followed by S-N-K’s *post hoc* comparison tests. *p* < .05 was regarded as significant and *p* < .01 was regarded highly significant.

## 3 Results

### 3.1 Anti-inflammatory assays

**FIGURE 2:**
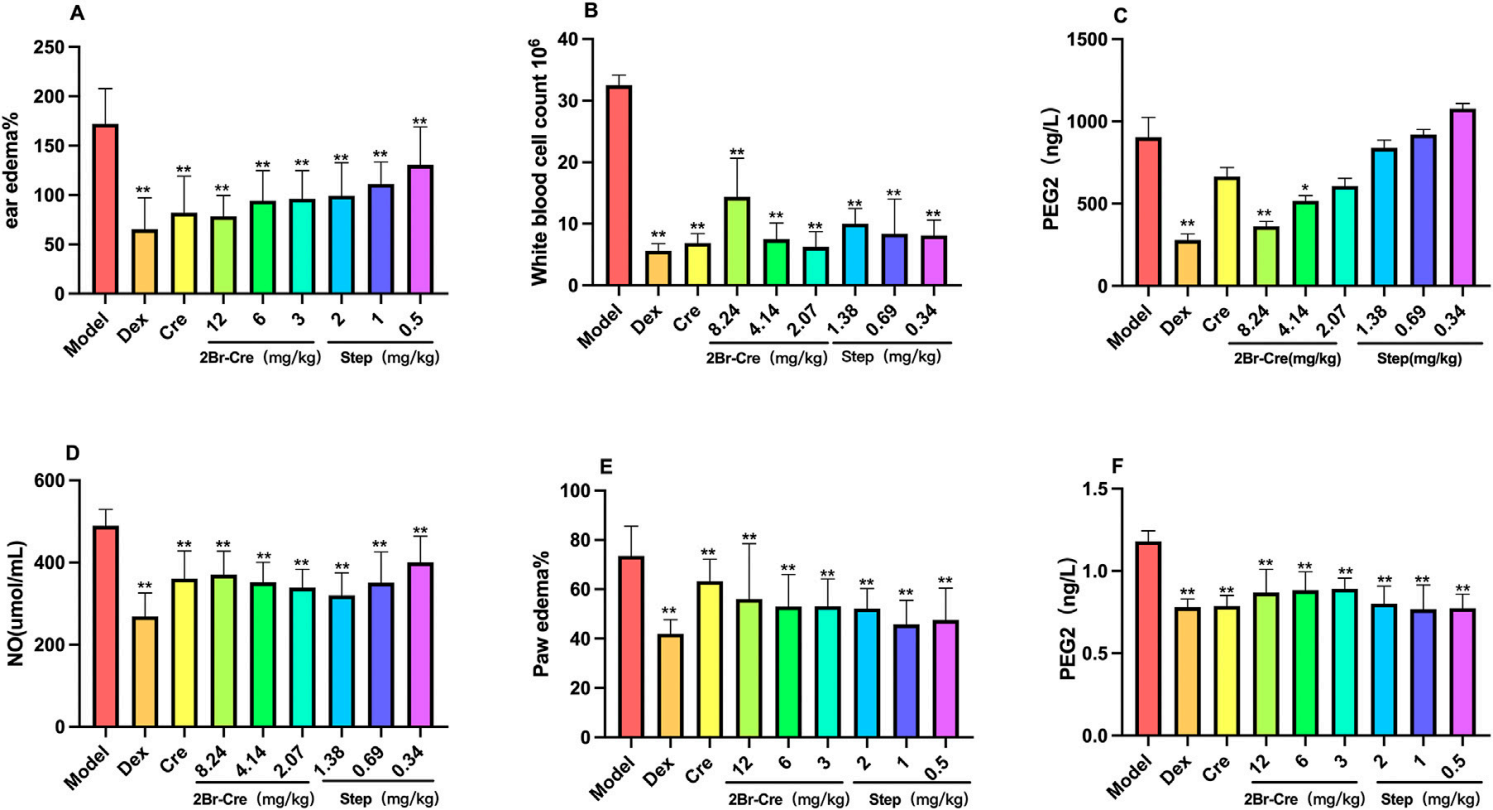
*In vivo* anti-inflammatory test results for 2Br-Cre and Step, **(A,E,F)**: The effects of 2Br-Cre (12, 6, and 3 mg/kg) and Step (2, 1, and 0.5 mg/kg) on xylene-induced ear edema and carrageenan-induced paw edema in mice; **(B,C,D)**: The effects of 2Br-Cre (8.24, 4.14, and 2.07 mg/kg) and Step (1.38, 0.69 and 0.34 mg/kg) on carrageenan-induced pleurisy in rats; Data are expressed as mean ± SD. *p < 0.05, **p < 0.01 *versus* Model group using one-way ANOVA followed by S-N-K’s *post hoc* multiple-comparison test.

Compared with the model group and the Dex group, different doses of 2Br-Cre and Step exerted significant inhibitory effects on xylene-induced ear edema in mice (F = 5.355, DFn = 7, DFd = 72, *p* < .01) in Figure 2A. Dex, Cre and all three doses of 2Br-Cre and Step significantly inhibited the number of white blood cells in pleural exudate (*p* < .01) in Figure 2B. The levels of PGE_2_ in exudate were significantly reduced at a high dose (*p* < .01) and medium dose (*p* < .05) of 2Br-Cre; however no inhibition of PGE_2_ levels were observed in the three doses of Step and Cre in Figure 2C. The levels of NO in pleural exudate were significantly reduced by Cre, the three doses of 2Br-Cre and the three doses of Step (*p* < .01) in Figure 2D on carrageenan-induced pleurisy. Compared with the model group and the Dex group, different doses of 2Br-Cre and Step had significant inhibitory effects on carrageenan-induced paw edema in mice (F = 5.969, DFn = 8, DFd = 81, *p* < .01) and significantly reduced the levels of PGE2 after inflammation (F = 15.197, DFn = 8, DFd = 81, *p* < .01 in Figure 2E, F.

### 3.2 Analgesic activities

#### 3.2.1 Hot plate testing and naloxone blocade

**FIGURE 3:**
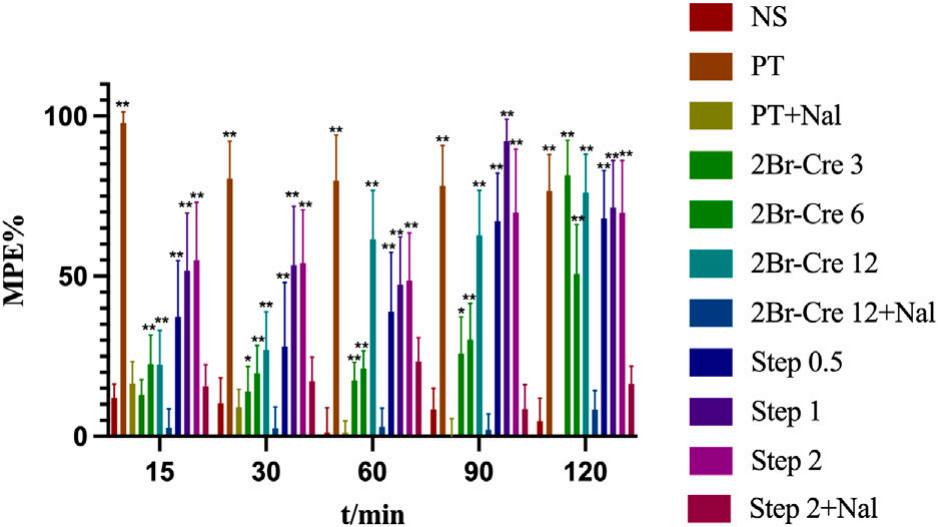
*In vivo* analgesic test results for 2Br-Cre and Step and reversal effect of naloxone in hot-plate test. Data are expressed as mean ± SD. *p < 0.05, **p < 0.01 *versus* NS using one-way ANOVA followed by S-N-K’s *post hoc* multiple-comparison test.

Compared with the NS group, the three doses of 2Br-Cre and Step significantly increased the pain threshold of mice affected by thermal stimulation (*p* < .05); In addition, there was a dose-effect relationship between Step and pain threshold. However, following naloxone antagonism, there was no significant change in pain threshold when compared between the administration group and the NS group, thus indicating that the analgesic effects of 2Br-Cre and Step could be blocked by naloxone. This finding indicated that the analgesic effects of 2Br-Cre and Step could be affected by opioid receptors. Results are shown in Figure 3.

#### 3.2.2 Writhing test

**FIGURE 4:**
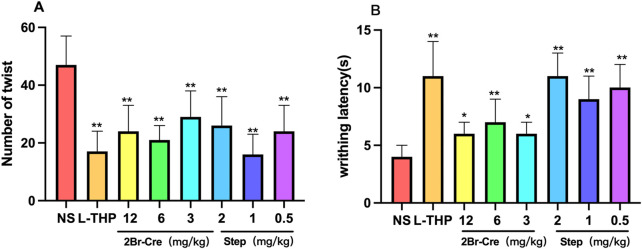
*In vivo* analgesic test results for 2Br-Cre and Step in Writhing test, **(A)** The effects of 2Br-Cre (12, 6, and 3 mg/kg) and Step (2, 1, and 0.5 mg/kg) on the number of acetic acid-induced writhing movements in mice. **(B)** The effects of 2Br-Cre (12, 6, and 3 mg/kg) and Step (2, 1, and 0.5 mg/kg) on acetic acid-induced first writhing latency in mice; Data are expressed as mean ± SD. *p < 0.05, **p < 0.01 *versus* NS group using one-way ANOVA followed by S-N-K’s *post hoc* multiple-comparison test.

Compared with the NS group, the three doses of 2Br-Cre and Step significantly inhibited the frequency of writhing in mice (F = 5.018, DFn = 14, DFd = 177, *p* < .05). Furthermore, the latency to the first writhe of mice was significantly prolonged in each administration group (F = 4.050, DFn = 14, DFd = 177, *p* < .05); however, there was no significant dose-effect relationship in any of the administration groups. Results are shown in Figure 4.

#### 3.2.3 CCI model experiment

**FIGURE 5:**
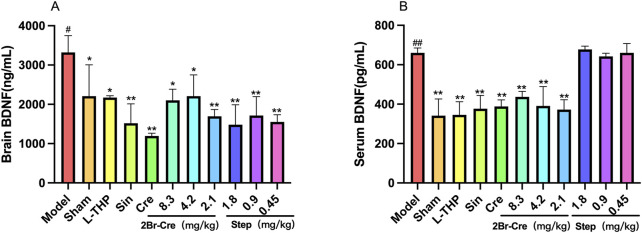
*In vivo* model of CCI test results for 2Br-Cre and Step. The effects of 2Br-Cre (8.3, 4.2 and 2.1 mg/kg) and Step (1.8, 0.9 and 0.45 mg/kg) on levels of brain **(A)** and serum **(B)** BDNF in rats; Data are expressed as mean ± SD. *p <0.05, **p <0.01 versus model group. ^##^ *p* <0.01 versus Sham group using one-way ANOVA followed by S-N-K’ s post hoc multiple-comparison test.

The PWMT of the Sham group did not differ significantly when compared before and after surgery. However, the PWMT of the other groups decreased significantly on the day 7 after surgery with significant difference when compared to that of the Sham group (*p* < .01). After successful model preparation, drug were continuously administered for 7 days. Then, the PWMT was carried out for 30 min on days 8, 10, 12 and 14. Compared with the Model group, the PWMT of each administration group was significantly increased (*p* < .01). As shown in Figure 5, Cre, Sino, 2Br-Cre and Step reduced the abnormal levels of BDNF in the serum and brain (*p* < .05), thus suggesting that the four compounds play an analgesic effect by simultaneously regulating the levels of central and peripheral BDNF. Results are shown in Table 1.

**TABLE 1:** PWMT of CCI rats at different times (‾x ± s, *n* = 10).

Group (mg/kg)	con	7 days	8 days	10 days	12 days	14 days
Model	42.6 ± 1.3	23.2 ± 2.4^##^	26.4 ± 3.2	29.5 ± 2.7	30.4 ± 3.8	31.3 ± 3.3
Sham	42.3 ± 0.7	42.2 ± 2	42.6 ± 4**	40.8 ± 1**	40.9 ± .3**	40.7 ± 2.1**
L-THP	44 ± 2.3	25.5 ± .7^##^	55 ± 2**	51.5 ± 3.4**	55.4 ± 2.1**	52.1 ± 2.4**
Sin	44.7 ± 1.1	23.9 ± 1.6^##^	37.3 ± 4.6**	42.6 ± 4**	44.6 ± 3.4**	45.9 ± 2.2**
Cre	44.2 ± 1.6	23.8 ± 1.2^##^	42.5 ± 3.5**	43.3 ± 1.8**	45.5 ± 2.6**	45.9 ± 2.6**
2Br-Cre 8.3	45.2 ± 3.7	26.3 ± 2.2^##^	50.1 ± 3.1**	52.6 ± 3.9**	49.1 ± 3.7**	45.4 ± 2.1**
2Br-Cre 4.2	41 ± 4	25.1 ± 4.6^##^	41.4 ± 2.9**	47.6 ± 3.2**	45.2 ± 1.6**	46 ± 4**
2Br-Cre 2.1	43.7 ± 4.3	24.3 ± 1^##^	42.2 ± 1.4**	43.2 ± 2.3**	40.9 ± 2.7**	39.8 ± 2.4**
Step 1.8	43.7 ± 1.3	24.5 ± 1.3^##^	41.2 ± 7.4**	50.1 ± 5.3**	50.3 ± 2.8**	49 ± 2.1**
Step 0.9	42.7 ± 11.9	23.5 ± 1.2^##^	46.9 ± 1.4**	45.9 ± 1.6**	44.5 ± 2.9**	43.6 ± 3.9**
Step .45	45.6 ± 1.8	24.4 ± 2.9^##^	40.7 ± 4.5**	43.7 ± 2.3**	42.4 ± 1.9**	40.4 ± 2.8**

Note: Data are expressed as mean ± SD. ^##^ *p* < .01, *versus* Sham group. *p < .05, **p < .01, *versus* Model group using one-way ANOVA, followed by S-N-K’s *post hoc* multiple-comparison test.

### 3.3 Cell experiments

#### 3.3.1 The inhibitory effects of 2Br-Cre and Step on NO and cytokines release from RAW264.7 macrophages induced by LPS

**FIGURE 6:**
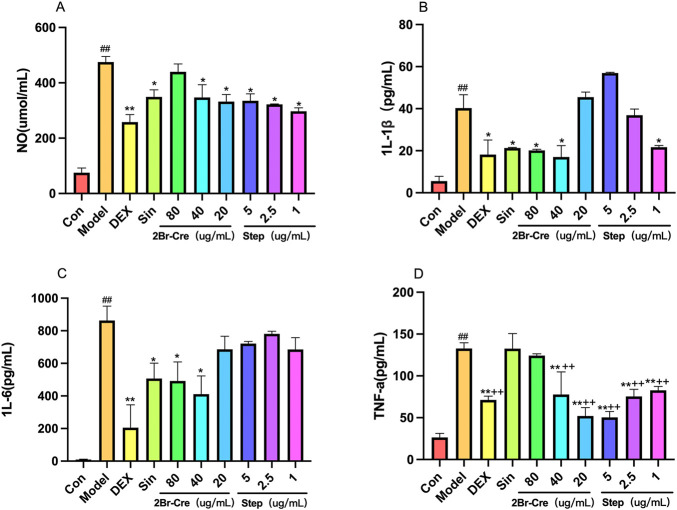
*In vitro* cell experiment results for 2Br-Cre and Step. The effects of 2Br-Cre (80, 40, and 20 μg/ml) and Step (5, 2.5, and 1 μg/ml) on proinflammatory cytokine release [TNF-α **(D)**, IL-1β **(B)**, and IL-6 **(C)**] and on nitric oxide **(A)** release by the RAW264.7 cells, Data are expressed as mean ± SD, (*n* = 3). *p <0.05, **p <0.01, versus Model group. ^##^ *p* <0.01 versus Con group. ^++^ *p* <0.01 versus Sin group using one-way ANOVA followed by S-N-K’s post hoc multiple-comparison test.

The medium and low doses of 2Br-Cre and the three doses of Step, significantly inhibited the release of NO in RAW264.7 cells induced by LPS (F = 1.056, DFn = 34, DFd = 4, *p* < .05). The medium and low doses of 2Br-Cre and the three doses of Step significantly inhibited TNF-α release (F = 36.152, DFn = 11, DFd = 24, *p* < .01) although the high dose group of 2Br-Cre had no significant inhibitory effect. The high and medium doses of 2Br-Cre and the low dose group of Step significantly inhibited IL-1β release (F = 6.272, DFn = 12, DFd = 26, *p* < .05), The high and medium doses of 2Br-Cre inhibited IL-6 release (F = 6.272, DFn = 12, DFd = 26, *p* < .05) although the low dose group of 2Br-Cre and the three doses of Step had no significant inhibitory effect. 2Br-Cre had different degrees of inhibition on the four inflammatory factors. These data indicate that the anti-inflammatory effects of Step may be achieved mainly by reducing the levels of TNF-α in Figure 6.

## 4 Discussion

After carrageenan is injected into the pleural cavity, the enzyme is activated to promote the release of some inflammatory factors, causing the inflammatory cells mainly neutrophils to gather and be activated to the inflammatory tissue (Wei, 2016). These neutrophils are mainly macrophages. Macrophages are activated, a series of important inflammatory factors related to inflammation will be released, such as TNF-α, PGE2, NO, which will induce a series of inflammatory reactions such as increased capillary permeability (Lee et al., 2016). PGE2 and NO come from two different inflammatory pathways respectively. Under the induction of inflammatory factors, arachidonic acid catalyzed the generation of PGE2 through the COX-2 pathway, while on the other side, MAPK, NF-κB and other signal channels activated to induce iNOSmRNA transcription l-arginine to produce a amount of NO (Kagemann et al., 2007; Yoon et al., 2015). Therefore, we can predict the mechanism by detecting the levels of NO and PGE2. 2Br-Cre and Step significantly inhibited the release of NO on carrageenan-induced pleurisy in rats, indicating that they affected MAKP, cGMP, and NF-κB signal transduction pathways to regulate the activity of nuclear transcription factor inhibitor IKB, and affect the expression of inflammatory factors. 2Br-Cre reduced the levels of PGE2 in the thoracic cavity of rats, reduced the levels of PGE2 in the exudate of inflammatory tissue of mice, and alleviated inflammatory exudation and edema. It was speculated that the anti-inflammatory mechanism of 2Br-Cre may be related to the inhibition of COX-2. Three doses of Step significantly inhibited the levels of PGE2 inflammatory tissue of mice, but there was no significant inhibitory effect on the levels of PGE2 on carrageenan-induced pleurisy in rats, and its mechanism needs to be further studied. In the present study, the selected doses is based on the LD50 of 2Br-Cre (LD50 = 59.62 mg/kg) (Wang et al., 2016) and Step (LD50 = 9.248 mg/kg). The dose and effect did not show a good dose dependence, mainly for the following two reasons: First, we tried to find the dose-effect range, but because the investigation of the lowest toxic dose and the lowest effective dose has not been carried out, there is no clear dose-effect relationship between the dose interval and the effect designed in our experiment, and the difference in effect has not been reflected, and the therapeutic index range has yet to be discussed. Second, the mechanism of action is not according to the dose-effect relationship. It remains to be further studied.

Nal is an opioid receptor antagonist, which can block the binding of PT to opioid receptors and antagonize the analgesic effect of PT. Studying the combination of drugs can reflect whether the mechanism of drug action is related to the opioid receptors, at least to a certain extent. The analgesic mechanism of L-THP occurs mainly by blocking dopamine receptors in the brain (Guo et al., 2014). Therefore, PT was selected as a positive control drug for antagonistic experiments involving naloxone. Analysis showed that the analgesic effect of PT was antagonized by Nal, thus indicating that the model had been successfully established. All of the groups without Nal antagonism shown that an increase in the threshold of heat pain. The analgesic effect of 2Br-Cre and Step on hot-induced pain was stable and their analgesic effects were antagonized by naloxone at 10 mg/kg, thus suggesting that 2Br-Cre and Step can exert central analgesic effects via opioid receptors. However, No antagonism was observed at 5 mg/kg (Arslan et al., 2010), both the dose and frequency of naloxone administration affected the duration of antagonism (Yano et al., 1979). The effects of dose and antagonism need further study. There are multiple subtypes of opioid receptors, including the *µ* receptor, *δ* receptor, *κ* receptor and opioid-like receptor norphin receptor; these are all associated with analgesia. In addition, drugs acting on opioid receptors, such as morphine and heroin are highly addictive. The specific analgesic effect of 2Br-Cre and Step depend on the subtype of receptor involved; further studies need to ascertain whether these drugs are weakly or strongly addictive.

Sin and L-THP are isoquinoline alkaloids belonging to the same genus as Cre and Step. Both Sin and L-THP are relatively effective drugs on the market. Therefore, they were selected as positive controls in this experiment. Cre, 2Br-Cre and Step are aporphine alkaloids with the same parent nucleus structure. The main difference between Cre and 2Br-Cre is mainly concentrated in ring D. After the introduction of a bromine atom, the LD50 of 2Br-Cre was more than six-fold higher than that of Cre, and the expected effect of toxicity reduction was achieved. Halogens are able to increase molecular lipophilicity, thus improving the permeability of lipid membranes; furthermore, the electronegativity of halogens can increase the biological activity of central molecules (Prabhakara et al., 2016). Previous studies have shown that the introduction of halogen atoms can still exert good physiological activity, such as the Heck reaction on sinomenine 1a while the introduction of halogen derivatives on ring A 1a leads to good anti-inflammatory activity. Following structural modification, lipid solubility is significantly increased; the lipid solubility of Cre is lower than that of 2Br-Cre. By comparing the analgesic effects of 2Br-Cre and Cre, we found that the effects of 2Br-Cre were superior to those of Cre in a nerve ligation model. Compared with previous reports, 2Br-Cre exhibited a longer effect on the pain threshold to heat than Cre, and its inhibitory effect on the frequency writhing caused by acetic acid was stronger than Cre.

Step and Cre are structural analogues and differ only in ring D 9c; there is one less methoxyl group in the 9c ring of Step than in Cre. The LD50 of Step and Cre are similar at 10 mg/kg. In this study, Step showed slightly better effects than Cre in the CCI behavioral experiment. This indicated that the methoxyl group in this position had no significant effect on the toxicity to aphophytes alkaloids. In addition, the structure of Step has better chemical stability than that of Cre. 2Br-Cre has the effect of detoxification and synergism through structural modification.

## 5 Conclusion

In the present study, both *in vitro and in vivo* experiments supported the fact that 2Br-Cre and Step exhibit anti-inflammatory and analgesic effects and that the underlying mechanism may be related to downregulation of the release of pro-inflammatory cytokines, thus providing a theoretical basis for future pharmaceutical research and development.

## Data Availability

The original contributions presented in the study are included in the article/supplementary material, further inquiries can be directed to the corresponding author.

## Glossary

### The following abbreviations are used in this document:

2Br-Cre: 10,11-dibrominecrebanine
5-HT: 5-hydroxy tryptamine
ANOVA: a one-way analysis of variance
BDNF: brain-derived neurotrophic factor
CCK-8: cell counting kit-8
Cre: crebanine
Dex: dexamethasone sodium phosphate injection
ELISA: enzyme-linked immunosorbent assay
FBS: fetal bovine serum
HPA axis: hypothalamic-pituitary-adrenal axis
IL-1β: interleukins-1β
IL-6: interleukins-6
LPS: lipopolysaccharide
L-THP: rotundine sulfate injection
Nal: naloxone
NO: nitric oxide
NS: normal saline
NSAIDs: non-steroidal anti-inflammatory drugs
PT: pethidine
Sin: zhengqingfengtongning injection
Step: stephanine
TNF-α: tumor necrosis factor-α
